# Microencapsulation Strategies in Veterinary Medicine: Overcoming Gastrointestinal Barriers in Monogastric and Ruminant Species

**DOI:** 10.3390/pharmaceutics18080944

**Published:** 2026-07-30

**Authors:** Milena de Gennaro, Vita D’Amico, Marianna Ivone, Annalisa Cutrignelli, Nunzio Denora, Angela Assunta Lopedota

**Affiliations:** Department of Pharmacy-Pharmaceutical Sciences, University of Bari Aldo Moro, 4, E. Orabona Street, 70125 Bari, Italy; milena.degennaro@uniba.it (M.d.G.); vita.damico@uniba.it (V.D.); marianna.ivone@uniba.it (M.I.); annalisa.cutrignelli@uniba.it (A.C.); nunzio.denora@uniba.it (N.D.)

**Keywords:** microencapsulation techniques, veterinary drug delivery, controlled release, monogastric and polygastric animals, feed additives, rumen-protected delivery, gastrointestinal stability

## Abstract

Oral delivery of bioactive compounds in veterinary medicine offers important opportunities to improve animal health, productivity, and welfare, but its effectiveness is often limited by species-specific gastrointestinal physiology. This review aims to provide a comprehensive overview of microencapsulation strategies for oral veterinary delivery, focusing on species-specific gastrointestinal barriers and the formulation approaches developed to overcome them. Monogastric and ruminant animals present distinct gastrointestinal environments that compromise the stability, bioavailability, and therapeutic performance of orally administered compounds. In monogastrics, gastric acidity, digestive enzymes, gastrointestinal transit, and microbiota-mediated interactions represent major barriers, whereas in ruminants, ruminal fermentation, prolonged retention, and physicochemical conditions may cause premature degradation of bioactive compounds. These barriers significantly hinder the successful use of probiotics, enzymes, essential oils, nutrients, vaccines, and veterinary drugs. However, microencapsulation has emerged as a promising solution, providing a protective barrier that improves compound stability, enhances gastrointestinal survival, and enables controlled or site-specific release. By preserving bioactivity and modulating release kinetics, microencapsulation contributes to improved delivery efficiency and functional performance. A distinctive feature of this review is the integration of species-specific gastrointestinal physiology with microencapsulation technologies to provide a rational framework for designing oral delivery systems in veterinary medicine, rather than focusing solely on individual encapsulation technologies. Current challenges related to material selection, formulation optimisation, and industrial scalability are discussed. Overall, this review highlights that integrating gastrointestinal physiology with advanced microencapsulation technologies is essential for developing effective, targeted, and sustainable oral delivery systems, ultimately supporting improved animal health, productivity, and welfare.

## 1. Introduction

Over time, oral administration has become one of the most widely used and practical routes for delivering bioactive compounds in veterinary medicine. This route offers several advantages, including ease of administration, reduced handling stress, improved animal compliance, and suitability for both individual treatments and large-scale livestock management. Orally administered compounds may include nutrients, probiotics, prebiotics, enzymes, phytochemicals, vaccines, antiparasitic agents, antibiotics, anti-inflammatory drugs, hormones, and other veterinary therapeutics. These substances are increasingly employed not only to treat diseases but also to improve animal health, productivity, welfare, feed efficiency, product quality, and the overall sustainability of modern animal production systems.

However, many bioactive compounds used in animal nutrition and veterinary medicine are highly sensitive to environmental and gastrointestinal (GI) conditions. Factors such as oxygen, moisture, light, heat, pH variations, digestive enzymes, and microbial metabolism may significantly compromise their stability, bioavailability, and biological efficacy during processing, storage, and GI transit. These GI barriers are particularly relevant for orally administered biologics and other sensitive bioactive compounds, highlighting the need for effective protective delivery systems [1]. In this context, microencapsulation has emerged as a versatile and powerful technology for the protection, stabilisation, and delivery of bioactive compounds across multiple scientific domains, including pharmaceuticals, food science [2] and veterinary applications. By entrapping active substances within a protective matrix or shell, microencapsulation enhances chemical stability, reduces degradation during processing and storage, and enables controlled or site-specific release [3]. The ability to achieve site-specific delivery largely depends on the physicochemical properties of the shell material, which can be tailored to respond to specific physiological stimuli encountered along the GI tract. Representative stimuli-responsive materials include pH-responsive polymers, such as Eudragit^®^ E, which dissolve under acidic conditions (pH < 5), enabling gastric release, and Eudragit^®^ L and S, which dissolve above pH 6.0 and 7.0, respectively, allowing site-specific release in different regions of the intestine. Enzyme-responsive materials, such as gelatin and protein- or starch-based matrices, are degraded by digestive enzymes, triggering the release of the encapsulated compound. Ion-responsive polymers, including ionically crosslinked alginate and pectin, undergo network relaxation following ion exchange with the surrounding medium, facilitating payload release. Finally, microbiota-responsive polysaccharides, such as pectin, guar gum, and dextran, are selectively degraded by enzymes produced by the colonic microbiota, enabling colon-targeted release. Therefore, the selection of the shell material should be guided by the physiological conditions of the target site and the desired release profile [4].

In veterinary applications, microencapsulation has gained increasing attention as a strategy to improve the delivery of nutritional and therapeutic compounds while supporting more sustainable animal production systems [5]. In addition, encapsulation technologies may improve palatability, facilitate feed incorporation, minimise undesirable interactions with feed components, and enable site-specific delivery.

However, the success of encapsulation strategies is closely linked to the physiological characteristics of the target species. Significant differences exist between monogastric animals, such as pigs and poultry, and polygastric, such as cattle and sheep, particularly in terms of GI structure, pH profiles, enzymatic activity, and microbial ecosystems. These differences critically influence the fate of encapsulated compounds, affecting their stability, release kinetics, and ultimate bioavailability [6]. A wide range of encapsulation materials and techniques has been investigated, including lipid-based systems, polysaccharide matrices, and protein-based carriers, each offering specific functional properties [7]. In monogastric animals, encapsulation is typically designed to protect bioactives from acidic gastric degradation and promote their release in the intestine, where absorption is optimal [8]. In contrast, ruminants present a unique challenge due to the presence of the rumen, a highly fermentative compartment rich in microbial activity, which can extensively degrade nutrients and bioactive compounds before they reach the lower GI tract.

In particular, the rumen represents a major challenge because microbial metabolism can extensively modify or degrade nutrients, bioactive compounds and veterinary drugs before they reach the lower GI tract, reducing their availability and functionality [9].

To address this issue, “rumen-protected” delivery systems have been developed to ensure that active compounds bypass or resist ruminal degradation and are released post-ruminally. Despite these advancements, several challenges and controversies remain. A key issue lies in balancing protection and release. This trade-off is particularly critical for rumen-protected formulations, where an optimal ruminal retention of approximately 60–80% is generally considered necessary to protect bioactive compounds during ruminal transit while ensuring their subsequent release and absorption post-ruminally. Excessively stable encapsulation systems may limit bioavailability, whereas insufficient protection can result in premature degradation. Furthermore, variability in experimental models particularly the inability of many in vitro rumen simulation systems to fully replicate the dynamic pH fluctuations, particle retention time, and microbial diversity observed in vivo, has contributed to inconsistent findings regarding the effectiveness of different encapsulation strategies across animal species. Economic feasibility, scalability, and regulatory considerations also represent significant barriers to the widespread adoption of microencapsulation technologies in veterinary practice.

In this context, the present review aims to provide a comprehensive and critical overview of microencapsulation strategies for veterinary applications, with a particular focus on how the physiological differences between monogastric and ruminant species influence the stability, release behaviour, and bioavailability of encapsulated bioactive compounds. The review compares the GI constraints affecting oral delivery in monogastric and ruminant animals, critically discusses the principal encapsulation materials and technologies currently employed in veterinary medicine, evaluates how species-specific GI conditions influence the performance of encapsulated bioactive compounds, and identifies the major limitations, research gaps, and future perspectives related to formulation optimisation, industrial scalability, in vivo validation, and regulatory implementation. By integrating these aspects, the review provides a species-oriented framework to support the rational design of encapsulation systems for veterinary applications, ultimately contributing to improved animal health, feed efficiency, product quality, and environmental sustainability.

## 2. Characteristics of Monogastric Animals: Digestive System Structure and Influences on Drug Release

**Figure 1:**
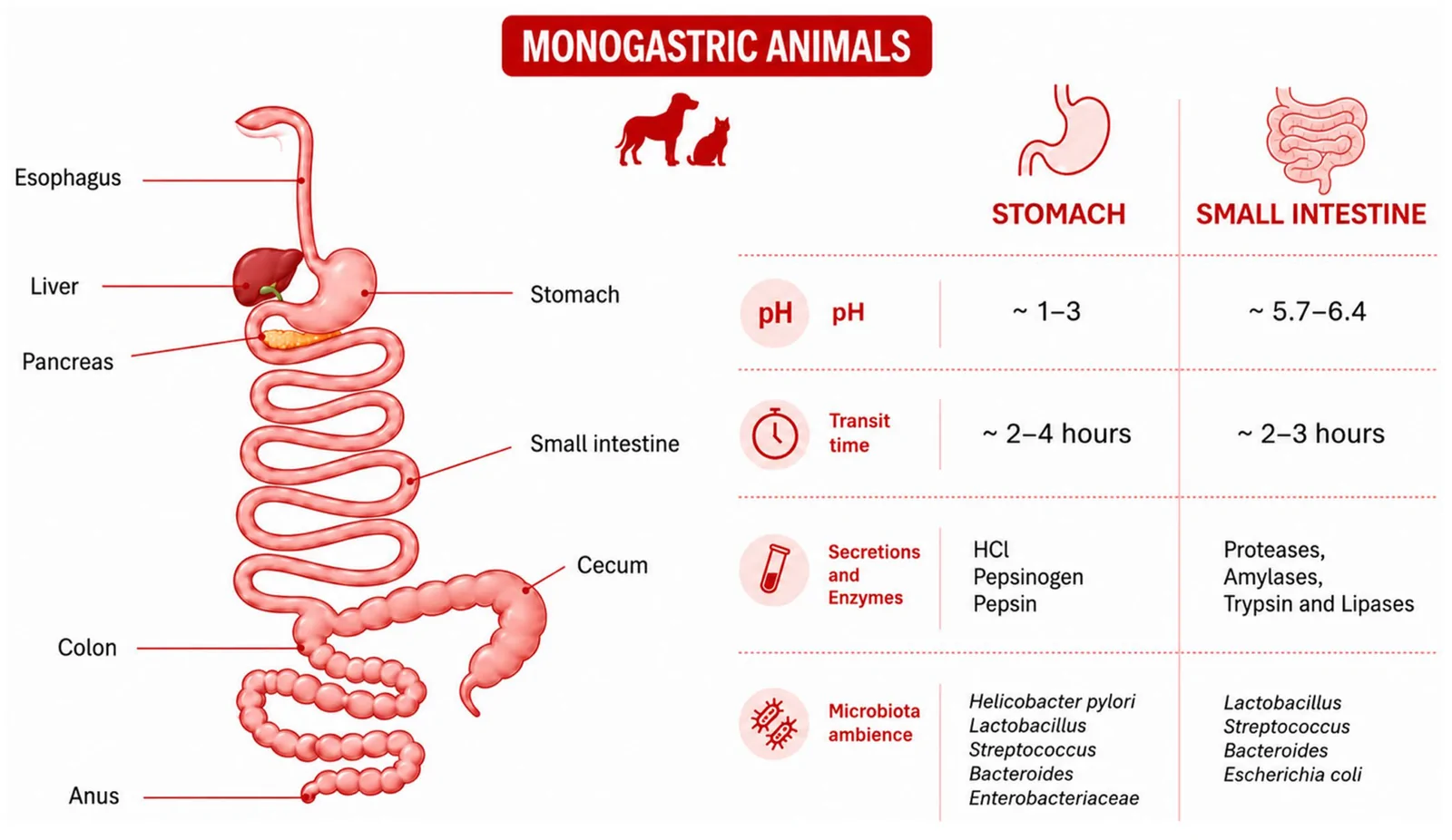
Monogastric GI anatomy and digestive environment.

Monogastric animals are vertebrates characterised by a digestive system composed of a single-chambered stomach (Figure 1), in which digestion occurs primarily through acid and enzymatic processes rather than through pre-gastric microbial fermentation associated with complex multi-compartmental systems typical of ruminants [10].

Following gastric digestion, nutrients are further hydrolysed and absorbed in the small intestine, which represents the major site of enzymatic digestion and nutrient uptake [11].

Despite sharing a common digestive organisation, monogastric species exhibit substantial differences in GI anatomy, digestive physiology, pH conditions, transit kinetics and microbial communities. These interspecies variations influence digestive efficiency and nutrient utilisation and therefore represent important considerations in veterinary nutrition and oral administration strategies [6,12].

### 2.1. Carnivorous Monogastrics

Dogs (*Canis lupus familiaris*) and cats (*Felis silvestris catus*) are characterised by a digestive system adapted to diets rich in animal-derived proteins. Their stomach exhibits intense hydrochloric acid secretion and high pepsin activity, with gastric pH values that may decrease to 1–2 [13]. Cats are considered obligate carnivores [13], whereas dogs have developed greater metabolic flexibility and improved carbohydrate utilisation through domestication and dietary adaptation [7]. The intestinal tract of these species is relatively short compared with that of herbivorous animals, reflecting a lower dependence on microbial fermentation. Nevertheless, the intestinal microbiota plays a fundamental role in metabolism, immune regulation, and gut homeostasis, with *Firmicutes*, *Bacteroidetes*, *Proteobacteria*, *Fusobacteria*, and *Actinobacteria* representing the dominant bacterial phyla [14,15].

### 2.2. Omnivorous Monogastrics

Pigs (*Sus scrofa domesticus*) possess a digestive system that closely resembles that of humans in terms of anatomy, digestive physiology, and microbiota composition, making them a widely used model in nutritional and pharmacological studies [16]. Digestion begins in the oral cavity and continues in the stomach, where hydrochloric acid and pepsin initiate protein degradation.

In adult pigs, gastric pH is highly acidic (2–4), supporting enzymatic activity and limiting pathogen survival. In contrast, pre-weaned piglets have lower acid secretion and a less acidic stomach due to milk-derived fermentation. After weaning, gastric acidity gradually increases, but transiently elevated pH (>5) can reduce protein digestion efficiency and increase susceptibility to disorders such as post-weaning diarrhoea [17].

The porcine intestinal microbiota is highly dynamic and contributes to nutrient metabolism, immune development and maintenance of intestinal integrity. *Prevotella*, *Bacteroides*, *Lactobacillus* and *Escherichia* are among the predominant bacterial genera, participating in the production of short-chain fatty acids (SCFAs) such as acetate, propionate and butyrate [16,18].

### 2.3. Avian Species

In avian species such as chickens and turkeys, the digestive system presents distinct anatomical specialisations that influence nutrient processing and oral delivery strategies. After ingestion, feed enters the crop, where temporary storage, hydration, and limited microbial fermentation occur, leading to the production of SCFAs such as lactate and acetate [19]. The feed subsequently reaches the proventriculus, where hydrochloric acid and pepsinogen initiate chemical digestion, and then the gizzard, which mechanically grinds ingested material and increases the surface area available for enzymatic digestion. The proventriculus has a strongly acidic pH (~2), while the gizzard shows slightly higher and variable values (2.6–4) depending on diet and retention time. The small intestine is specialised in enzymatic digestion and absorption, whereas the ceca are the main site of microbial fermentation of undigested substrates [20].

Microbial density is low in the small intestine but high in the ceca, where fermentation is more active. The cecal microbiota is mainly composed of Firmicutes and Bacteroidetes, with contributions from Proteobacteria and Actinobacteria. Key genera include *Lactobacillus* in proximal regions and *Ruminococcus*, *Bacteroides*, *Clostridium*, and *Faecalibacterium* in the ceca [21,22].

### 2.4. Herbivorous Monogastric

In herbivorous monogastric animals such as horses (*Equus ferus caballus*) and rabbits (*Oryctolagus cuniculus*), the GI tract is adapted for extensive microbial fermentation of plant fiber, compensating for the absence of foregut fermentation.

In horses, the stomach is relatively small and mainly serves as a storage and initial digestion site, while most fermentation occurs in the cecum and colon, which make up over 60% of the digestive tract and host an active anaerobic microbiota with a near-neutral pH (6.3–7.5) [23,24].

In rabbits, the cecum is even more developed and plays a central role in fiber digestion. They also perform cecotrophy, the reingestion of soft feces rich in microbial biomass and nutrients, which enhances dietary efficiency [25,26].

In both species, the hindgut microbiota is dominated by Firmicutes and Bacteroidetes, including fiber-degrading bacteria such as *Lachnospiraceae*, *Ruminococcaceae*, and *Prevotellaceae*. In horses, maintaining microbial balance is crucial to prevent dysbiosis-related disorders such as colic and hindgut acidosis [27,28].

## 3. Characteristics of Polygastric Animals: Multi-Compartment Digestive System (Rumen, Reticulum, Omasum, Abomasum) and Influence on the Release of Substances

**Figure 2:**
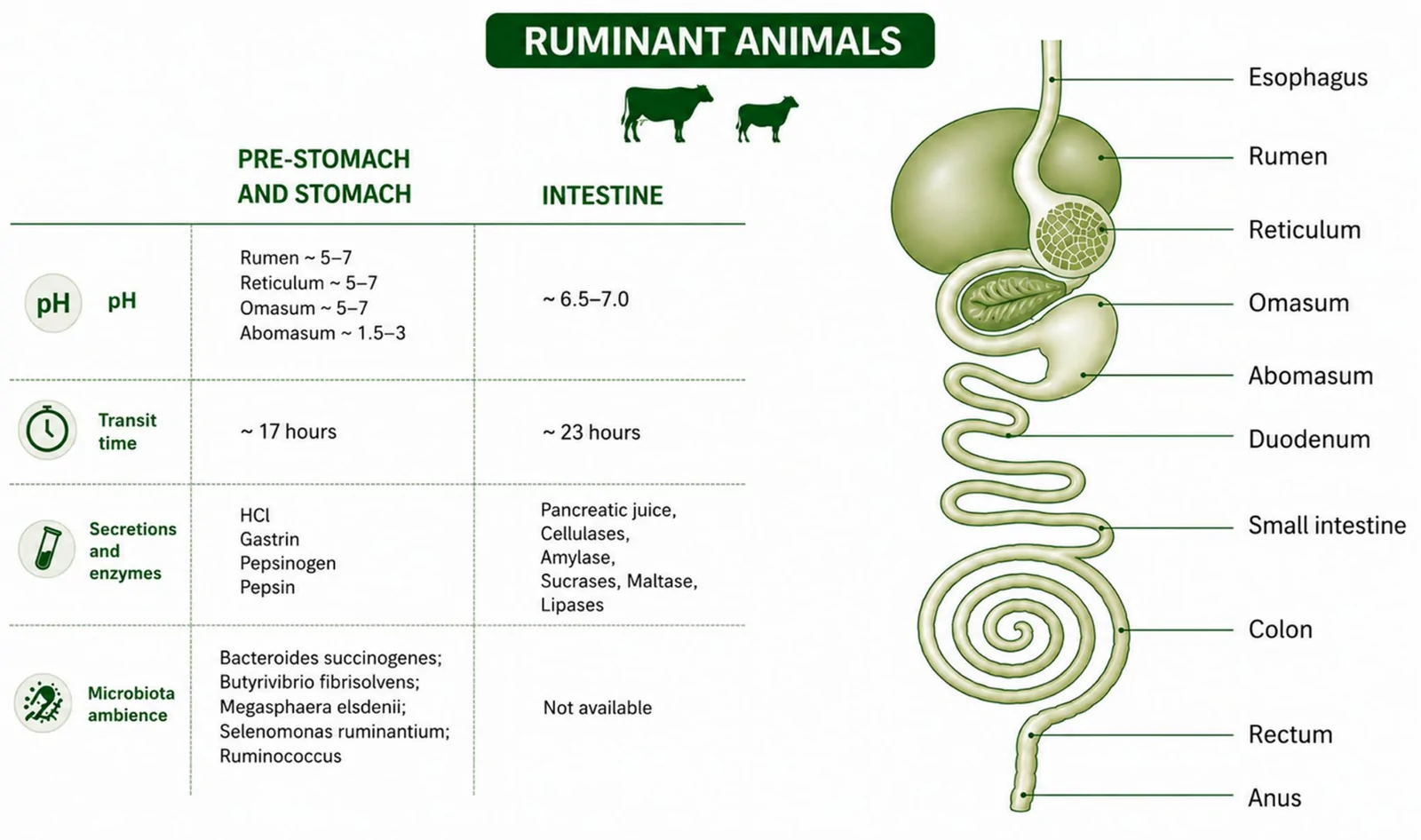
Polygastric GI anatomy and digestive environment.

Ruminants are herbivorous polygastric animals characterised by a highly specialised digestive system composed of four anatomically and functionally distinct compartments: the rumen, reticulum, omasum and abomasum (Figure 2) [29]. This multi-compartment organisation enables efficient utilisation of fibrous plant materials through pregastric microbial fermentation, allowing ruminants to derive energy from structural carbohydrates that monogastric animals cannot digest effectively.

### 3.1. Rumen

The rumen is the largest digestive compartment and serves as the principal site of microbial fermentation. In adult cattle, its volume may exceed 100–150 L, providing a stable anaerobic environment that supports dense microbial populations composed of bacteria, archaea, protozoa and fungi [30]. This diverse microbial ecosystem degrades complex polysaccharides such as cellulose and hemicellulose, converting them into volatile fatty acids (VFAs), primarily acetate, propionate and butyrate, which serve as the principal energy source for the host [31,32]. Approximately 70–90% of VFAs are absorbed directly across the ruminal epithelium via passive diffusion and transporter-mediated mechanisms [33,34]. Fermentation efficiency is enhanced by rumination, which reduces particle size and increases substrate surface area available for microbial colonisation [35]. The ruminal epithelium exhibits considerable adaptive plasticity, including papillae remodelling and regulation of transport systems such as MCT1, optimising VFA absorption in response to dietary composition [36,37].

In addition to carbohydrate metabolism, the rumen also contributes to nitrogen recycling through microbial utilisation of urea transferred from blood and saliva [38,39]. Rumen digesta dynamics are characterised by selective particle retention and regulated passage. Larger particles are retained for further fermentation, whereas smaller particles and soluble fractions pass more rapidly toward the omasum. Retention time may vary considerably depending on diet composition and particle size, directly influencing fermentation kinetics and digestive efficiency [40]. However, the physicochemical characteristics of the ruminal environment, including pH fluctuations and continuous microbial activity, strongly influence digestive processes and nutrient transformation [41].

### 3.2. Reticulum

Closely associated with the rumen, the reticulum functions as a sorting and mixing chamber. Its honeycomb structure facilitates the separation of particles according to size and density, directing larger particles back toward the rumen for further fermentation while allowing smaller particles and fluids to progress distally. The coordinated motility of rumen and reticulum (reticulorumen contractions) ensures effective mixing, fermentation and regurgitation during rumination [42].

### 3.3. Omasum

The omasum, characterised by numerous laminae or “leaves,” plays an important role in the absorption of water, electrolytes and residual VFAs before digesta enters the abomasum. Its structure increases surface area and contributes to the regulation of fluid flow toward the lower GI tract [43]. By concentrating digesta and regulating passage rate, the omasum contributes to maintaining optimal fermentative conditions in the rumen.

### 3.4. Abomasum

The abomasum, often referred to as the “true stomach,” resembles the monogastric stomach both structurally and functionally. It secretes hydrochloric acid and digestive enzymes such as pepsin, initiating acid-mediated digestion of microbial protein and residual feed components. This compartment marks the transition from microbial fermentation to enzymatic digestion before nutrient absorption in the small intestine [42].

## 4. Microencapsulation

Microencapsulation is a rapidly emerging technology that involves coating small liquid or solid capsules with a polymeric film. It is used to turn liquids into solids, change surface and colloidal features, preserve the environment, and regulate the release characteristics of different coated materials [44]. The diameters of capsules vary from 1 to 1000 µm, and they can be utilised to encapsulate various substances such as enzymes, medications, adhesives, flavourings, agricultural products, and live cells [45]. Microencapsulation has found applications in pharmaceutical, food, agricultural and veterinary fields due to its ability to protect sensitive compounds and modulate their release under specific conditions [46].

### 4.1. Microencapsulation in the Veterinary Area

The applications of microencapsulation in the veterinary field have expanded significantly in recent years, driven by the increasing demand for more effective and species-specific oral delivery systems for bioactive compounds administered to both livestock and companion animals [47]. Current veterinary applications encompass nutraceuticals, feed additives, probiotics, vaccines, and therapeutic agents, highlighting the versatility of microencapsulation across different fields [48].

**Figure 3:**
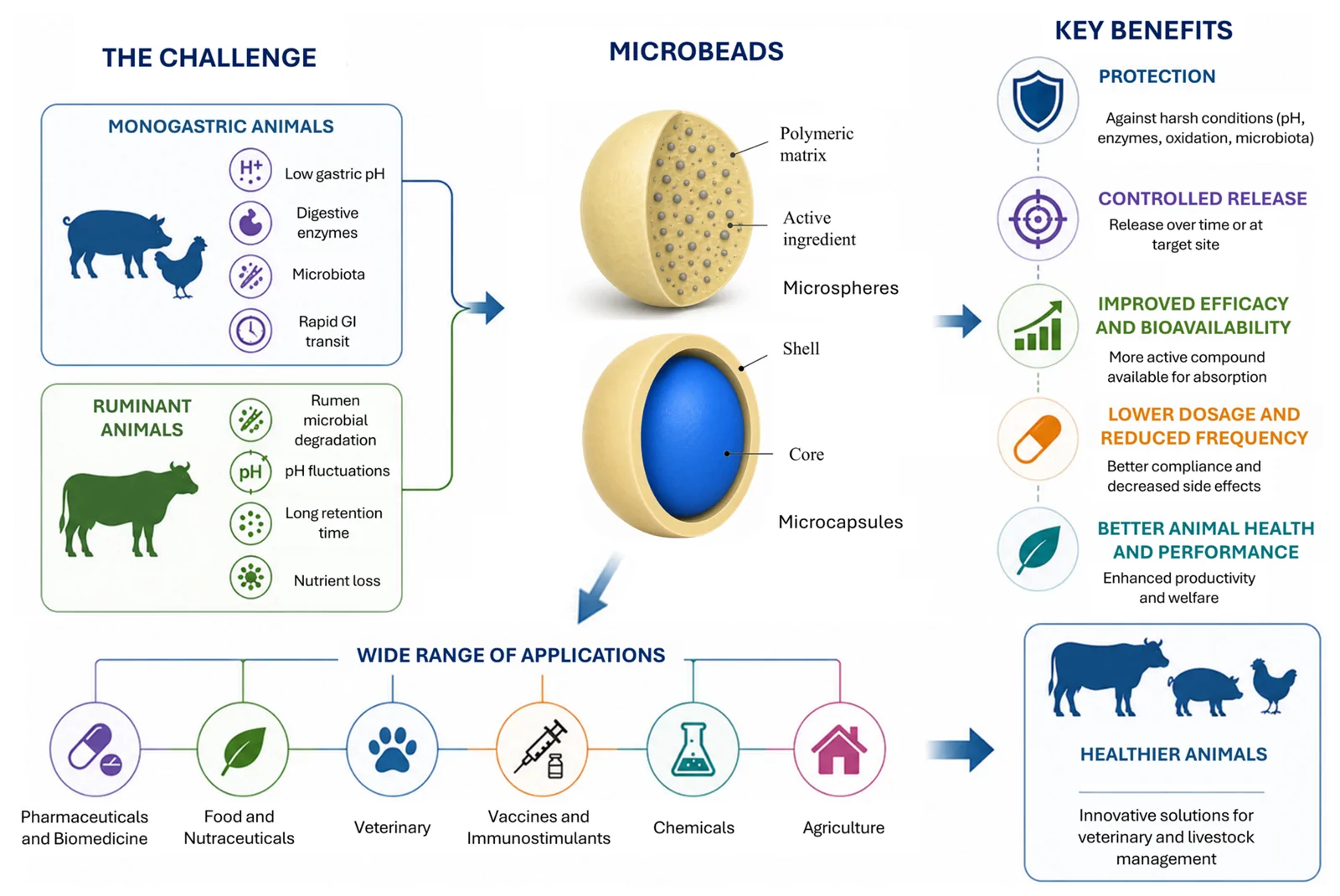
Reasons for microencapsulation in the veterinary field.

One of the main areas of use is in feed supplements. Microencapsulation is widely used to deliver vitamins, minerals, essential amino acids such as lysine and methionine, fatty acids and essential oils (*Citrus essential oil*), protecting them from oxidation and degradation during food processing or GI transit (Figure 3). In addition, microencapsulation facilitates the transformation of liquid substances into stable, free-flowing powders suitable for feed incorporation [49], while masking unpleasant tastes and odours and reducing hygroscopicity, thereby improving palatability and voluntary intake [50,51]. From an economic point of view, these advantages translate into optimised farm management, reduced treatment costs, and improved feed efficiency [52].

The administration of probiotics and prebiotics represents another important field of application. Microencapsulation ensures microbial viability during feed processing, storage and passage through the gastric environment, enabling the release of live microorganisms in the intestinal tract, where they exert beneficial effects on host health. Probiotic strains like *Lactobacillus plantarum*, *Bifidobacterium* spp. and *Lactobacillus* spp. can be delivered within protective matrices resistant to acid conditions and digestive enzymes, an approach particularly relevant in young animals or those exposed to stress factors such as antibiotic treatments or dietary changes.

Microencapsulation has also demonstrated significant potential in the vaccine sector. Encapsulation of antigens and immunostimulatory adjuvants has been shown to improve vaccine formulation and enhance immune responses [53]. Chen et al. [54] investigated PLGA microspheres loaded with recombinant staphylococcal enterotoxin A as a single-dose vaccine for immunisation against *S. aureus* using a mouse model. Negash et al. [55] also explored the properties of PLGA microbeads as a mucosal delivery carrier of deoxyribonucleic acid (DNA) vaccine based on virus protein 2 (VP2), which is a neutralising antibody inducer, aimed at protecting chickens from the infectious bursal disease virus (IBDV). In addition, the authors proposed the association of plasmid-encoded chicken interleukin-2 (chIL-2) or CpG-ODN, aiming to improve DNA vaccine efficacy. Furthermore, the possibility of administering vaccines orally and in sustained-release form helps to reduce the number of booster shots, improving adherence to vaccination protocols and reducing handling stress [56,57].

In therapeutic treatments, microencapsulation is used for the controlled release of antiparasitic agents such as ivermectin and moxidectin [58], as well as anti-inflammatory, antimicrobial and antifungal drugs, including chlorhexidine [59]. These formulations may improve dosing strategies and therapeutic performance while reducing fluctuations in systemic drug exposure. Although less extensively investigated than therapeutic drug formulations, microencapsulation has also been explored for veterinary insecticides and ectoparasiticides. The primary objective of these systems is to prolong residual efficacy through controlled release while improving formulation stability and, in some cases, reducing treatment frequency, operator exposure, and environmental dispersion [60]. Early studies demonstrated that microencapsulated permethrin provided significantly longer residual activity against *Stomoxys calcitrans* in lactating dairy cows than conventional formulations, maintaining effective insecticidal activity on the hair coat for up to seven days after treatment [61]. More recently, gel-based microencapsulated pyrethroid formulations containing permethrin and α-cypermethrin have shown promising efficacy against tick infestations in dairy cattle, suggesting that encapsulation may improve the persistence of topical ectoparasiticides under field conditions [62]. In addition, polyurea microcapsules containing diazinon have been successfully incorporated into disinfectant formulations intended for livestock facilities, providing controlled insecticide release while improving the physicochemical stability of combined disinfectant–insecticide formulations [60]. Despite these encouraging results, the application of microencapsulation to veterinary insecticides and ectoparasiticides remains relatively limited, with most studies still confined to experimental or proof-of-concept formulations.

Similar principles apply to the delivery of hormones such as progesterone and oestradiol for oestrus synchronisation, as well as to growth-promoting implants and wound-healing formulations incorporating antibacterial agents and tissue regeneration promoters [63].

Overall, these diverse applications highlight the versatility of microencapsulation as a platform technology capable of addressing nutritional, therapeutic, and management needs across veterinary medicine.

### 4.2. Rationale for Microencapsulation in Monogastric Animal Formulations

The oral delivery of bioactive compounds in monogastric animals is challenged by several physiological barriers. Gastric acidity represents one of the main constraints, particularly in carnivorous species and poultry, where low pH conditions may compromise the stability of acid-sensitive molecules [64,65].

In addition, digestive enzymes can promote the premature degradation of proteins, peptides and other susceptible compounds before they reach their absorption site [66].

The intestinal microbiota may further influence compound fate through metabolic transformation or inactivation of bioactive molecules and xenobiotics [67,68].

As a consequence, sensitive ingredients such as probiotics, enzymes, organic acids, essential oils and bioactive peptides may exhibit reduced biological efficacy when administered orally [69,70].

Therefore, encapsulation strategies for monogastric species should primarily focus on protecting acid- and enzyme-sensitive compounds during GI transit while ensuring their release at the desired intestinal site [71,72,73,74,75].

### 4.3. Rationale for Microencapsulation in Ruminant Animal Formulations

In ruminant animals, oral administration of bioactive compounds is intrinsically complex due to the highly active microbial environment of the rumen. Proteolytic and deaminative microorganisms rapidly hydrolyse dietary proteins and free amino acids, while microbial lipases and biohydrogenating bacteria modify unsaturated fatty acids and other lipophilic compounds, markedly reducing their post-ruminal availability and biological functionality [76].

Consequently, prolonged ruminal residence increases the likelihood of degradation or biotransformation of orally administered compounds before they reach the small intestine [77,78]. These limitations are particularly relevant for compounds intended to exert their activity beyond the rumen. Essential amino acids such as methionine and lysine undergo extensive microbial breakdown, markedly reducing their intestinal availability [79,80]. Likewise, trace minerals may exhibit reduced bioavailability due to interactions occurring within the ruminal environment [81,82]. Functional additives and certain veterinary drugs can also be susceptible to microbial modification or premature biotransformation, potentially compromising their intended efficacy [83,84].

Collectively, these physiological and microbiological constraints underline the importance of considering ruminal metabolism as a key factor during the design of oral veterinary formulations.

## 5. Microencapsulation Techniques in the Veterinary Area

**Figure 4:**
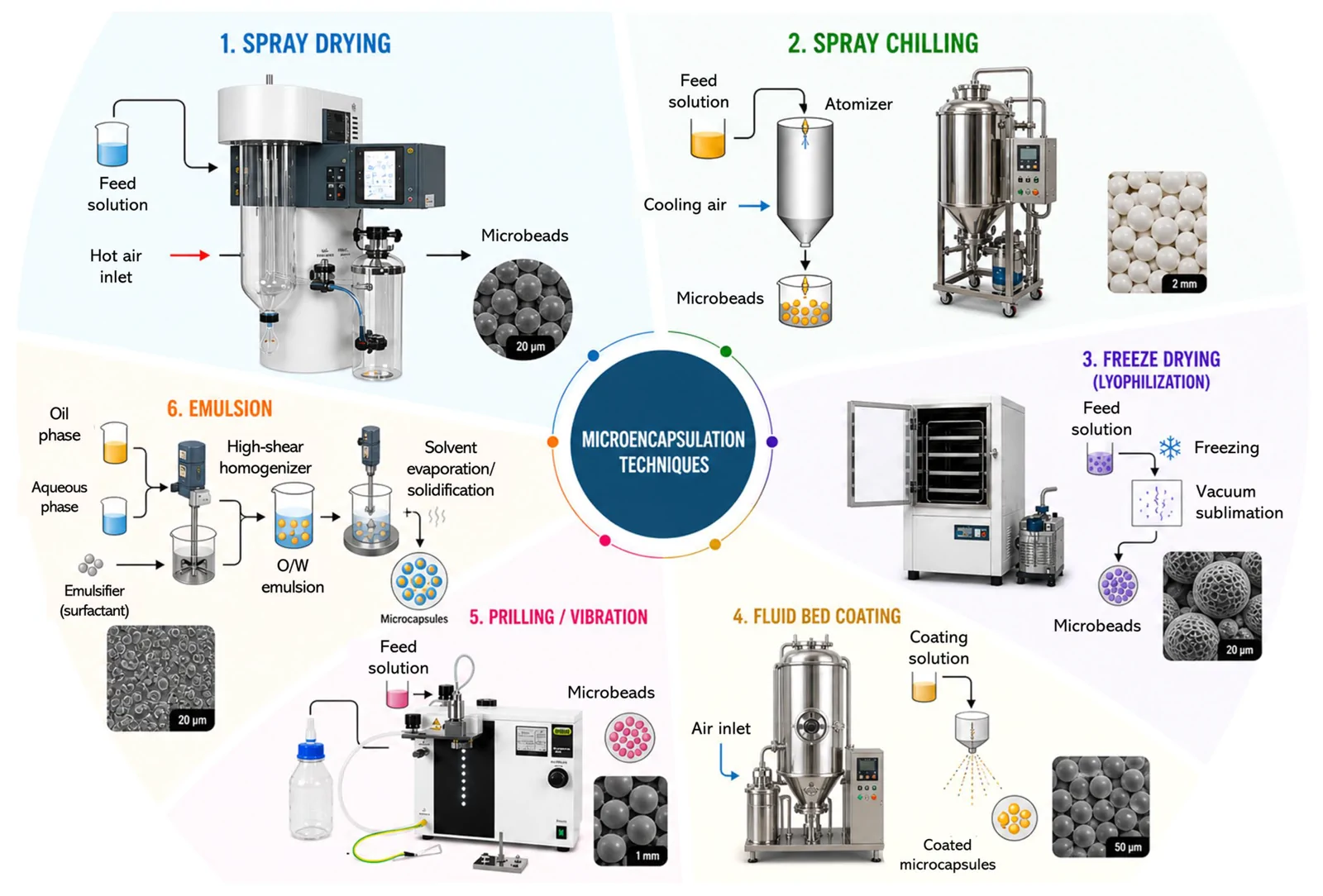
Microencapsulation techniques.

Various microencapsulation techniques are available, including extrusion, emulsion, fluidised bed, freeze drying, spray-freezing, spray chilling and electrospraying (Figure 4). Microencapsulation is a fundamental technique in veterinary medicine for improving the stability, bioavailability and controlled release of active ingredients and nutrients intended for animals. The choice of method depends on the physical and chemical properties of the material to be encapsulated and the coating, as well as the area of application of the microcapsules. This review will provide a critical evaluation of the main microencapsulation techniques used in veterinary medicine, highlighting their advantages and applications.

### 5.1. Spray Drying

Microencapsulation by spray drying is an economical and versatile technology widely used in the food, pharmaceutical, chemical and veterinary sectors, particularly in animal nutrition [85]. It allows liquids, suspensions or solutions to be transformed into fine powders through a continuous and reliable process, which can be carried out using widely available equipment.

The process involves preparing a feedstock fluid obtained by mixing the bioactive ingredient with a coating material (encapsulating agent). The encapsulating material should be selected according to the physicochemical properties of the active ingredient and the intended application [86]. After homogenisation, the mixture is atomised using nozzles or rotating discs, generating droplets which, when in contact with a flow of hot air, quickly lose the solvent, forming dry particles. These are then separated from the drying gas using a cyclone and collected. The use of two- or three-fluid nozzles allows for the production of matrix microparticles or core–shell microcapsules, respectively, by modulating the technology according to specific application requirements. Spray drying generally produces nearly spherical microparticles with diameters ranging from approximately 1 to 50 μm, although particle size depends on formulation characteristics, such as solids content and viscosity, as well as process parameters, including atomisation conditions and drying temperature.

Microencapsulation using spray dryers is widely used in veterinary medicine and animal nutrition thanks to its versatility and numerous advantages. In veterinary medicine and animal nutrition, spray drying is particularly attractive because it enables the large-scale production of stable powdered formulations suitable for incorporation into feed or pharmaceutical products. Depending on the formulation, spray-dried microparticles can preserve sensitive ingredients during processing and GI transit, allowing their release at the desired site of action [87]. In addition, it facilitates the transformation of liquid products into easily manageable powders and allows for targeted release to specific tissues, also promoting reproductive synchronisation in farm animals.

Despite its many advantages, this technology has some limitations in the veterinary field. Coating materials must have optimal water solubility to ensure effective processing, thus limiting the range of polymers that can be used. Furthermore, substances that are poorly soluble or unstable in aqueous formulations are difficult to process, reducing the versatility of the method. This necessitates careful selection and optimisation of formulations, with possible repercussions on the cost and effectiveness of the final product.

Nevertheless, spray drying remains one of the most widespread and advantageous methods for microencapsulation in animal husbandry, thanks to its simplicity, cost-effectiveness and versatility. Several studies have confirmed its effectiveness in improving the delivery and functionality of nutritional compounds in animal production systems. For example, methionine, an essential amino acid for farm animals, has been encapsulated using spray drying with a mixture of gelatin and sodium alginate. Different ratios between methionine and the mixture were tested, and the microcapsules obtained in this way were shown to modulate the release of methionine, also promoting the absorption of other amino acids. Furthermore, in vivo, encapsulation reduced the degradation of methionine in the rumen and increased muscle mass in cattle, suggesting greater intestinal availability of the amino acid [88]. Similar studies have shown that encapsulated lysine and DL-methionine also improve nutritional efficiency in broiler chickens [89]. These findings highlight the relevance of spray drying for the ruminal bypass of essential amino acids with well-documented economic, nutritional and environmental benefits [90,91].

Another example concerns hydrolysed casein, which has been encapsulated in maltodextrin by spray drying to mitigate the typical unpleasant taste [92].

As regards probiotics, it has been demonstrated that *Lactobacillus plantarum* can be microencapsulated with mixtures of fructo-oligosaccharides (FOSs), whey protein isolate and denatured proteins, ensuring high encapsulation efficiency, reduced residual moisture, improved stability during storage and greater resistance to simulated gastric and intestinal pH conditions [93].

Similar probiotic applications have also been reported in ruminant nutrition. For instance, *Lactobacillus* spp. encapsulated with gum arabic and maltodextrin exhibited high stability and encapsulation efficiency, supporting its use as a functional feed additive for beef cattle [94]. Beyond probiotic formulations, spray drying has also been applied to the encapsulation of microbial biomass and biologically active by-products [95]. In this context, spray-dried spent brewer’s yeast has demonstrated high stability and functionality when used as a supplement for dairy cows, contributing to nutrient recycling and circular feed approaches [96]. Other reported applications include the encapsulation of ruminal fluids with enzymatic potential, obtained from post-slaughter waste, using materials such as sodium alginate, guar gum, chitosan and maltodextrin [97]. In addition to biological additives, encapsulated zinc oxide has been shown to increase average daily gain and reduce feed consumption in weaned piglets [98].

Spray-drying applications have also been extended to phytogenic compounds, where encapsulation of *Minthostachys verticillata* essential oil and limonene preserved chemical integrity and demonstrated adjuvant potential in bovine mastitis vaccine formulations [99]. Encapsulated essential oils, produced through spray-drying, have demonstrated the ability to modulate ruminal fermentation and reduce gas production in vitro, suggesting potential applications in methane mitigation strategies for ruminants [100,101].

Beyond nutritional applications, spray-drying has also been employed in veterinary pharmacology to achieve controlled release and enhanced stability of therapeutic compounds. In this regard, progesterone microparticles produced with chitosan–TPP by spray-drying demonstrated high encapsulation efficiency and release kinetics suitable for estrus synchronisation in cattle [102]. More recently, the technique has been applied to stabilise thermosensitive vaccine components, further emphasising its potential to extend beyond nutrition to broader aspects of livestock health management [103].

Overall, spray drying can be regarded as the most established microencapsulation technology currently available for veterinary applications. Unlike several other encapsulation approaches that are still largely confined to experimental studies, spray drying has already demonstrated successful translation to commercial feed additives and pharmaceutical formulations. Nevertheless, most published studies remain focused on formulation development and physicochemical characterisation, while comparative in vivo evidence demonstrating clear superiority over alternative encapsulation technologies is still limited.

Representative examples of spray-dried veterinary formulations are summarised in Table 1, highlighting the broad applicability of this technology to nutrients, probiotics, phytogenic compounds, vaccines and therapeutic agents.

### 5.2. Spray Chilling

Spray chilling, also known as spray congealing or spray cooling, is a lipid microencapsulation technique that is well established in the pharmaceutical and food industries and is now also rapidly expanding in the veterinary field. It involves dispersing the active ingredient in a melted lipid carrier, such as hydrogenated oils, cocoa butter or other food/pharmaceutical fats, and then atomising the mixture through a heated nozzle in a cooling chamber [105]. When exposed to cold air or liquid nitrogen, rapid heat transfer causes the lipid to crystallise and form solid spherical microparticles [106,107,108,109]. Larger particles are collected at the base of the chamber, while finer particles are recovered using a cyclone. The technique is particularly attractive for thermolabile compounds, furthermore, it avoids the use of organic solvents and exposure to high processing temperatures, while offering good scalability and relatively low production costs [110,111]. Spray chilling generally produces spherical lipid microparticles with diameters ranging from approximately 20 to 200 μm, although the final particle size depends on formulation characteristics, particularly the lipid composition and melt viscosity, as well as atomisation conditions and cooling rate.

In animal husbandry, spray chilling is establishing itself as a versatile technology for the delivery of different classes of bioactive compounds. Extensively studied for oxygen-sensitive anaerobic probiotics, such as *B. animalis* subsp. *lactis*, it allows for the preservation of vitality greater than 80–90% post production and over 50% after 120 days at 4 °C [112]. Lipid matrices protect anaerobic probiotics by creating an oxygen-impermeable barrier; upon ingestion, these matrices are degraded by digestive lipases, facilitating the gradual release of viable cells into the gut [113]. Consistent with these findings, single- and double-layered capsules containing *B. bifidum* BB-12, obtained by using hydrogenated palm oil, presented an encapsulation efficiency of 92% and survival rates of 88% to 75% during gastric and intestinal digestion, respectively [114].

A well-established application of spray-chilling in ruminants is the production of rumen-protected amino acids. Mepron^®^, a commercial spray-cooled methionine formulation, has demonstrated improved post-ruminal methionine availability and enhanced milk protein synthesis in dairy cows, validating the efficacy of lipid-based encapsulation systems in bypassing ruminal degradation [115]. Comparable benefits have been reported for spray-chilled lysine, where intestinal absorption and nitrogen efficiency improved relative to unprotected sources [79]. Additional applications include digestive enzymes, vitamins, polyunsaturated fatty acids and essential oils, for which lipid encapsulation improves stability and protection against degradation during storage and administration [112].

Spray-chilling has also been explored for the encapsulation of organic acids and plant-based additives, aiming to stabilize functional compounds and modulate rumen fermentation. Lipid-encapsulated citric acid improved metabolic responses and fiber digestibility in dairy cattle, suggesting that protected organic acids may enhance microbial efficiency and overall rumen function [116]. Although direct applications of spray-chilled phytogenic additives in ruminants remain limited, recent studies indicate that lipid-based encapsulation can effectively stabilize essential oils and control their release in feed systems, highlighting a promising strategy for future rumen-bypass applications [117].

Spray chilling has also shown considerable potential in monogastric animals. For example, in weaned piglets, essential oils and organic acids such as cinnamaldehyde, carvacrol, and thymol were microencapsulated in stearic acid lipid matrices by spraying and rapid solidification into microcapsules. The administration of these microcapsules improved intestinal health, reduced post-weaning diarrhea, and increased production performance compared to non-encapsulated formulations, highlighting the potential of microencapsulation to modulate the release of bioactive compounds in monogastric animals [74].

Finally, the technology has been proposed for the oral administration of vaccines and antigens, thanks to the protection offered by lipid particles against adverse GI conditions [107].

In contrast to spray drying, spray chilling cannot yet be considered a mature microencapsulation technology for veterinary applications. Although it offers clear advantages for heat-sensitive compounds, the available evidence is largely restricted to feed additives and functional ingredients, with few examples of translation to veterinary pharmaceuticals or commercial products. Consequently, its current value lies primarily in niche applications where thermal protection is prioritised over sophisticated release control.

### 5.3. Freeze Drying

Freeze-drying consists of three stages: freezing, primary drying through ice sublimation, and secondary drying to remove residual moisture [118]. Similar to spray chilling, freeze-drying preserves the structural and functional integrity of heat-sensitive compounds, making it particularly suitable for biological materials and other labile ingredients.

Among its veterinary applications, freeze-drying has emerged as one of the most effective strategies for the stabilisation of microorganisms and biological products. Probiotics have been microencapsulated using this technique to increase their survival during storage [119]. More recently, alginate-based microencapsulation combined with freeze-drying has been shown to significantly improve the storage stability, GI tolerance, and antimicrobial activity of *Saccharomyces boulardii*, further supporting the potential of this approach for probiotic delivery in poultry production [120]. In ruminant nutrition, lyophilised *Bifidobacterium* spp. administered to neonatal calves maintained intestinal colonisation for over two months and markedly reduced faecal *Escherichia coli* and coliforms compared with untreated animals [121]. Likewise, *Limosilactobacillus reuteri* SW23 delivered in a freeze-dried microencapsulated form improved average daily gain, feed efficiency and beneficial lactic acid bacteria counts more effectively than non-encapsulated or air-dried preparations in dairy calves [122].

More recently, this approach has been extended to the stabilisation of complex microbial consortia, as Islam et al. (2024) demonstrated that freeze-dried faecal microbiota preparations improved clinical recovery and donor microbial engraftment in neonatal calves suffering from diarrhoea compared with frozen formulations, reinforcing the potential of lyophilisation for microbiome-based therapeutics in ruminants [123]. Even in companion animals, such as cats, microencapsulation through freeze-drying has improved the stability of probiotics and intestinal colonisation, promoting wider applications in the field of veterinary nutraceuticals [71].

Beyond probiotics and microbial supplements, freeze-drying is also a key technology for vaccine stabilisation in the bovine sector, particularly in regions where effective cold-chain management is challenging. A combined freeze-dried vaccine against Rift Valley fever and bovine ephemeral fever demonstrated strong immunogenicity and extended storage stability in cattle [124] while a multivalent lyophilised formulation targeting BVDV-1/2, BoHV-1, BRSV and PI3V retained antigenic integrity and induced robust humoral responses in calves [125].

In addition to biological products, freeze-drying has also been applied to the encapsulation of nutrients and other bioactive compounds. For example, Ma et al. [126] encapsulated a whey protein hydrolysate with sodium alginate or a mixture with whey proteins to reduce bitterness and improve the hygroscopic stability of the product, while maintaining its immunomodulatory function intact.

Freeze-drying has also been applied to the encapsulation of oils, as in the case of linseed oil treated with zein [127].

Current veterinary evidence indicates that freeze-drying has established a highly specialised rather than broadly applicable role in microencapsulation. Most studies consistently focus on preserving the viability and functionality of biological systems, including probiotics, microbial consortia and vaccines, whereas comparatively few applications involve conventional drugs or nutritional additives. This suggests that the main strength of freeze-drying lies in maintaining biological activity rather than expanding the range of encapsulated veterinary products.

### 5.4. Fluid Bed Coating

Fluid bed coating is a well-established technology for microencapsulating drugs and nutrients in the veterinary field. The process is based on suspending solid particles (active ingredients or nutritional additives) in a stream of hot air that keeps them in continuous motion, creating a “fluid bed”. A solution containing the coating material, generally consisting of natural polymers (cellulose, starch), proteins or gums [128], is sprayed onto these particles. The rapid evaporation of the solvent results in the formation of a solid, uniform layer that encapsulates the active ingredient. Since fluid bed coating is applied to preformed particles or pellets, the final particle size is largely determined by the dimensions of the starting cores and the thickness of the coating layer.

In veterinary applications, fluid bed coating is primarily employed to protect bioactive compounds and modulate their release through the deposition of functional coating layers tailored to specific physiological targets.

Among the most established applications in ruminant production systems is the formulation of rumen-protected amino acids. The commercial product Smartamine^®^ M exemplifies this technology: according to its original patent (US4675175A), methionine granules are coated with pH-sensitive ethylcellulose and stearic acid layers using fluid bed techniques, ensuring strong resistance to ruminal degradation while enabling rapid dissolution under abomasal acidic conditions. In vivo evidence supports the efficacy of this approach, as supplementation with Smartamine^®^ M increased milk yield and plasma methionine concentrations in high-producing dairy cows under tropical conditions [129]. More recent evaluations reinforce the relevance of rumen-protected amino acid technologies. Räisänen et al. (2025) assessed the bioavailability of protected histidine, lysine and two methionine products produced through different coating strategies, reporting relative bioavailability values ranging from approximately 45% to 91%, depending on the formulation method [130]. These findings highlight how fluid bed coating enables precise nutritional delivery in ruminants, supporting both productive performance and amino acid efficiency.

Beyond nutritional applications, fluid bed coating has also been explored for the formulation of biologically active compounds intended for oral delivery. In particular, this technology has been applied to the production of coated vaccine microspheres for porcine epidemic diarrhoea virus (PEDV). In this approach, antigen-loaded microspheres were coated using a fluidized bed process to protect the vaccine from GI degradation, enabling effective oral administration. In vivo studies in weaned piglets demonstrated the induction of PEDV-specific immune responses, highlighting the potential of fluid bed coating for the development of oral vaccines in monogastric species [131].

Current evidence suggests that the main strength of fluid bed coating lies in the precise engineering of functional coating layers rather than in the encapsulation process itself. Most veterinary applications exploit this technology to tailor the interaction between the formulation and the GI environment, particularly for rumen-protected products. Consequently, its success depends less on the active ingredient than on the design and performance of the coating material, making coating selection the key determinant of product efficacy.

### 5.5. Nozzle Extrusion Techniques (Prilling/Vibration Technique)

The extrusion technique is a physical method, the oldest and often preferred method to microencapsulate. There are several technologies to produce microbeads by extruding a liquid through a nozzle or orifice, which are based on different mechanisms for breaking up the liquid jet: conventional dripping (simple extrusion), electrostatic cutting, jet cutter or mechanical cutting technology with a rotating disc or jet cutting, vibrating nozzle [132]. Among these approaches, the prilling/vibration technique has gained increasing attention for veterinary and biotechnological applications. The prilling/vibration technique is based on breaking a laminar jet of polymer solution into a line of one-dimensional droplets using a vibrating nozzle device. To prevent droplet coalescence, an electrostatic charge is typically applied on the droplet surface, after which the droplets fall into a consolidation bath, where they solidify into microbeads. This system enables the production of microcapsules or microspheres with a narrow and well-controlled size distribution, a key advantage for reproducible delivery systems [133,134,135]. Depending on the nozzle diameter, vibration frequency, flow rate, and formulation properties, the prilling/vibration technique generally produces highly monodisperse microcapsules or microspheres with diameters typically ranging from approximately 200 to 2000 μm, although both smaller and larger particles can be obtained under specific processing conditions.

The prilling/vibration method is a technique that does not require high temperatures or solvents, ensuring high cell viability [136]. The process is efficient, rapid, reproducible, and industrially scalable [137,138]. It also allows the use of natural polymers such as alginates, pectin, carrageenans or gelatins, which are biocompatible and safe.

In the veterinary field, extrusion-based prilling techniques have been extensively investigated for the encapsulation of live microorganisms, aiming to improve their stability, controlled release and biological efficacy. *Lactobacillus lactis* and *Bifidobacterium bifidum* were microencapsulated using a multilayer matrix composed of phosphatidylcholine and vitamin E, lactose and gum arabic, and maltodextrin to evaluate their effect on the serum biochemical parameters of broiler chickens [139]. In pigs, dietary supplementation with these encapsulated probiotics resulted in significant improvements in serological parameters, including increased total protein, globulins and plasma albumin concentrations, along with reduced cholesterol levels. Beyond microbial delivery, prilling-based extrusion techniques have also been applied to the encapsulation of volatile and sensitive bioactive compounds. For example, in a study [140], the encapsulation of volatile aromatic ingredients, such as carvacrol, was described using extrusion, employing glassy matrices based on carbohydrates (sucrose, maltodextrin, glucose syrup, glycerine and glucose) capable of conferring high stability to the compound.

In subsequent studies [141], carvacrol was microencapsulated with a combination of alginate and whey protein, obtaining microcapsules capable of allowing complete release of the compound within four hours in the intestines of pigs.

Recent findings further extend the application of this technique to ruminant production systems. A comprehensive review by Almassri et al. (2024) emphasised the growing importance of microencapsulation technologies for protecting enzymes and sensitive nutrients from ruminal degradation and ensuring their targeted release at the intestinal level [47]. Preliminary experimental studies have further demonstrated the feasibility of producing enzyme-loaded alginate beads using vibrating-nozzle systems, supporting the potential of this technology for rumen-protected enzyme delivery. Current evidence suggests that the main value of the prilling/vibration technique lies in its formulation flexibility rather than in a specific veterinary application. The ability to process natural polymers under mild conditions makes this technology particularly attractive for sensitive biological systems, yet most reported applications remain at the experimental stage. Consequently, future progress will depend less on demonstrating encapsulation feasibility—which has already been widely established—than on validating reproducible in vivo performance and facilitating translation into practical veterinary formulations.

### 5.6. Emulsion Technique

Microencapsulation using emulsion technology is based on the dispersion of two or more immiscible liquid phases, within which the active ingredient, usually in liquid form, is dispersed in the continuous phase containing a dissolved polymer. Subsequent modifications of the two-phase system led to the formation of a protective layer around the dispersed droplets, which solidifies to form micro-delivery systems, depending on the formulation strategy and processing conditions [142]. The stability of the emulsion is strongly influenced by the selection of surfactants, which reduce the interfacial tension between the dispersed and continuous phases and prevent droplet coalescence. Surfactants are selected according to their hydrophilic-lipophilic balance (HLB), with low-HLB surfactants generally used to stabilise water-in-oil (W/O) emulsions and high-HLB surfactants preferred for oil-in-water (O/W) systems. In addition, the use of solid lipids, liquid lipids, or mixtures of both may influence the distribution of the encapsulated bioactive within the matrix, thereby affecting encapsulation efficiency, particle morphology, and release behaviour. Following emulsification, the subsequent solidification of the polymeric phase leads to the formation of protective microcapsules surrounding the dispersed droplets. Emulsion-based techniques generally yield particles with diameters ranging from approximately 1 to 500 μm. The final particle size, size distribution, and encapsulation performance are determined by the emulsion type, polymer composition, homogenisation conditions, and subsequent solidification process, which collectively influence particle morphology and release characteristics. Several technological variants of emulsion-based microencapsulation have been developed to tailor particle structure and release behaviour. Among the most widely used approaches is emulsification followed by solvent evaporation, where solvent removal induces polymer precipitation around the active compound, generating polymeric microparticles. This method has been used to encapsulate immunogenic proteins (LpanUA.27.1860 and LpanUA.22.1260) in PLGA-based particulate systems for the development of an experimental vaccine formulation against leishmaniasis [143].

Another important variant is emulsification followed by chemical or ionic cross-linking, which is particularly suitable for sensitive compounds and living microorganisms [144]. In the field of animal husbandry, emulsion followed by cross-linking has found increasing application in the microencapsulation of probiotics for animal feed. In particular, Rodklongtan et al. and Zhang et al. have shown that microencapsulation of probiotics by emulsion and subsequent cross-linking increases the survival of microorganisms during gastric transit, protecting them from acidic conditions and the action of digestive enzymes. Encapsulated probiotics also showed improved modulation of the intestinal microbiota compared with non-encapsulated forms [72,75].

Methionine has been microencapsulated in carnauba wax to allow its release in the intestines of ruminants, preventing degradation in the rumen [145]. Similarly, urea, a non-protein nitrogen compound, has been encapsulated in carnauba wax to achieve gradual release in the rumen, thereby avoiding toxic ammonia peaks and optimising nitrogen utilisation by the ruminal microbiota [146]. Eugenol, thymol, vanillin and rosemary essential oil have also been effectively encapsulated using oil-in-water emulsions stabilised by Maillard reaction products between soy proteins and polysaccharides. The particles obtained showed good stability and, in in vivo tests, did not alter the qualitative characteristics of the meat in the treated pigs, nor the levels of muscle collagen, supporting their suitability for use in animal feed [147].

Growing evidence highlights the relevance of emulsion technologies in ruminant nutrition and health. Ibrahim and Hassen (2021) demonstrated that *Acacia mearnsii* tannins incorporated into palm- and sunflower-oil matrices achieved high encapsulation efficiency and gradual release under simulated rumen and intestinal conditions, confirming the suitability of oil-based emulsions for protecting and targeting polyphenolic compounds [84]. Similarly, Budiman et al. (2024) developed a double-layer peanut-oil emulsion that reduced lipid degradability and modified fermentation dynamics in an in-vitro rumen model, indicating the potential of multilayer emulsions to limit premature lipolysis and modulate microbial ecosystems [148].

Complementing these findings, Adejoro et al. (2018) produced tannin-loaded lipid microparticles using a solid-in-oil-in-water technique, achieving reduced ruminal gas production while preserving bioactive functionality, demonstrating how emulsified encapsulation can influence fermentative processes without compromising efficacy [149].

These findings are consistent with recent evidence highlighting microencapsulation as a promising strategy to protect rumen-targeted feed additives, modulate the ruminal microbiome, and contribute to enteric methane mitigation while maintaining the biological activity of encapsulated compounds [150].

The main strength of emulsion-based microencapsulation is its remarkable formulation versatility. However, this flexibility also represents a major limitation, as small changes in formulation or processing conditions can substantially affect particle characteristics and reproducibility. Consequently, successful applications rely on careful formulation optimisation rather than the encapsulation technique itself.

## 6. Limitations and Challenges of Microencapsulation in Veterinary Applications

Despite the numerous advantages discussed so far, microencapsulation technologies still present several limitations and formulation challenges in veterinary applications. A comparative overview of the main advantages, disadvantages associated with the most used techniques is reported in Table 2. The development of effective microencapsulated systems requires careful control of formulation and process parameters, as environmental factors such as oxygen, humidity, temperature and mechanical stress may compromise the stability of encapsulated compounds, particularly biological materials and living microorganisms [151]. In addition, industrial-scale production may involve significant costs associated with specialised equipment, process optimisation and quality control procedures, particularly when maintaining the biological activity of sensitive compounds throughout manufacturing and storage.

The selection of coating materials also remains challenging, as no single material simultaneously provides optimal mechanical resistance, permeability, stability, biodegradability and cost-effectiveness. For example, in ruminant applications, insufficient coating resistance may result in premature ruminal degradation and reduced intestinal delivery [152]. Another major limitation concerns the translation of experimental results into practical applications. Although many formulations show promising performance under in vitro conditions, their behaviour may differ substantially in vivo due to the complexity and variability of GI and ruminal environments [47]. Research gaps are particularly evident in ruminant systems, where studies on the encapsulation of enzymes, functional microorganisms and other sensitive bioactives remain relatively limited compared with those conducted in monogastric species. Furthermore, limited disclosure of formulation details in commercial products often hinders direct comparison between technologies and reduces the reproducibility of published findings [153]. The long-term biological effects of some encapsulated bioactives, particularly complex microbial formulations, also require further investigation to ensure safety and efficacy under field conditions [154]. An additional challenge is the marked variability among animal species. Differences in GI physiology, metabolism, feeding behaviour, microbial populations and environmental conditions often require species-specific formulation strategies, limiting the universal applicability of individual delivery systems [155]. For example, pH-responsive coatings should be selected according to the GI pH profile of the target species, allowing rumen-stable formulations to remain intact under the near-neutral conditions of the rumen while releasing their payload in the acidic abomasum, whereas formulations for monogastric species, such as pigs, are generally designed to withstand gastric conditions and release the active ingredient in the small intestine. Likewise, release kinetics should be adapted to species-specific GI transit times, with prolonged protection required during ruminal retention and more rapid release profiles often needed in poultry due to their considerably shorter GI transit. Furthermore, differences in microbial populations and digestive enzyme activity among species may influence the degradation of encapsulating materials and the stability of bioactive compounds, requiring the selection of appropriate wall materials to ensure effective protection and targeted release.

Finally, route-specific limitations should also be considered. Oral delivery systems may be affected by feed selectivity, voluntary feed refusal, chewing behaviour and interspecies differences in GI physiology, all of which can influence the performance of microencapsulated formulations [156]. Similarly, topical and mucosal delivery systems may face challenges related to retention at the administration site, animal behaviour and local tolerability [157].

**Table 2:** Advantages and disadvantages of the main microencapsulation techniques used in veterinary applications.

Techniques	Wall Materials	Advantages	Disadvantages	Ref.
Spray drying	Phthalate, modified starch, soy protein isolates	High production efficiency; easy powder handling; simple process; low cost; reproducible; short processing time	Limited wall materials; variable particle size; not suitable for thermolabile compounds; oxidative degradation; nonuniform particles; material loss during drying	[85]
Spray chilling	Hydrogenated oils, cocoa butter, lipid matrices, stearic acid	Low operation cost; suitable for heat-sensitive compounds; solvent-free process; controlled release; easy scale-up	Process parameter sensitivity; rapid release of actives; limited to hydrophobic compounds; nonuniform particles	[107]
Freeze drying	Maltodextrins, sorbitol, gums, trehalose	Low operating temperature; suitable for temperature-sensitive compounds; minimal core material damage; high product stability; good product quality	Slow process; high production cost; expensive equipment; difficult particle size control; porous particle structure; post-processing sieving required	[158]
Fluid bed coating	Casein, alginate, waxes	Uniform particle size; controlled release; good coating uniformity; high encapsulation efficiency; scalable process	Particle fragility; low production efficiency; multiple influencing parameters	[128]
Nozzle extrusion techniques (prilling/vibration technique)	Alginate, calcium chloride, gellan gum, protein	Good sealing properties; long storage stability; cost-effective process; no high temperatures required; no organic solvents required; mild processing conditions; good barrier properties	High production cost; high shear stress; variable particle size and shape; difficult processing of viscous solutions; core material instability	[133]
Emulsion technique	Gum arabic, gelatin, chitosan	High stability; Suitable for temperature-sensitive compounds; Mild processing conditions; Good protection of active compounds; Controlled release	Low production efficiency; difficult process control; variable particle size; possible emulsion instability	[159]

## 7. Conclusions

In conclusion, this review highlights the pivotal role of microencapsulation in addressing the main challenges associated with oral administration in veterinary medicine. Due to the significant differences in GI physiology between monogastric and ruminant animals, orally administered bioactive compounds are exposed to distinct anatomical, biochemical, and microbiological environments that strongly influence formulation performance. Consequently, successful encapsulation strategies should be designed according to the physiological characteristics of the target species.

This review provides a comprehensive overview of the principal microencapsulation materials and technologies currently investigated for veterinary applications, critically discussing their advantages, limitations, and suitability for different classes of bioactive compounds. The comparative analysis presented in this review highlights that no single encapsulation strategy is universally applicable. Instead, the selection of encapsulation materials and technologies should be guided by the physicochemical properties of the bioactive compound, the target species, and the intended site of action.

However, the challenge of effectively scaling these technologies for industrial veterinary applications remains significant, requiring a careful balance between production costs, encapsulation efficiency, stability, and large-scale manufacturing feasibility. The future of veterinary microencapsulation technologies will depend on overcoming these challenges and creating innovative, sustainable, and highly effective solutions for modern animal production and therapeutic management.

## Figures and Tables

**Table 1:** Examples of spray-dried microencapsulation systems for veterinary applications.

Encapsulated Active	Encapsulating Material(s)	Application	Main Outcome	Ref.
Methionine	Gelatin + sodium alginate	Ruminal bypass of essential amino acids	Reduced ruminal degradation and increased muscle mass in cattle	[88]
Lysine and DL-methionine	Stearic acid matrix	Poultry nutrition	Improved nutritional efficiency in broiler chickens Improvement of amino acid utilisation efficiency in broiler chickens	[89]
Hydrolysed casein	Maltodextrin	Feed palatability improvement	Reduced unpleasant taste	[92]
*Lactobacillus plantarum*	FOS + whey protein isolate + denatured proteins	Probiotic delivery	Improved stability and GI resistance	[93]
*Lactobacillus* spp.	Gum arabic + maltodextrin	Functional feed additive	High encapsulation efficiency and storage stability	[94]
Ruminal fluids with enzymatic activity	Alginate + guar gum + chitosan + maltodextrin	Recovery of bioactive slaughterhouse by-products	Successful encapsulation of enzymatically active fluids	[97]
Zinc oxide	Lipid-encapsulated ZnO (Shield Zn^®^)	Piglet supplementation Growth promotion and intestinal health support in weanling pigs	Improved average daily gain compared to native ZnO at the same physiological level; enhanced growth performance and reduced post-weaning intestinal stress	[98]
Essential oils (*M. verticillata*, limonene)	HPMC + maltodextrin	Adjuvant strategy for bovine mastitis vaccines	Preserved chemical integrity and biological activity	[99]
Progesterone	Chitosan–TPP	Estrus synchronisation in cattle	Controlled release and high encapsulation efficiency	[102]
Albendazole + Praziquantel	Eudragit^®^ EPO + beeswax	Taste masking of veterinary antiparasitic drugs	Reduced drug release at salivary pH while maintaining release at gastric pH; improved spray-drying yield	[104]
Thermosensitive vaccine components	Spray-dried stabilising matrix (not specified)	Stabilisation of veterinary vaccine formulations	Improved thermal stability and preservation of vaccine component functionality during storage	[103]

## Data Availability

No new data were created or analyzed in this study. Data sharing is not applicable to this article.
